# The identification of potent dual-target monopolar spindle 1 (MPS1) and histone deacetylase 8 (HDAC8) inhibitors through pharmacophore modeling, molecular docking, molecular dynamics simulations, and biological evaluation

**DOI:** 10.3389/fphar.2024.1454523

**Published:** 2024-09-16

**Authors:** Huilian Hua, Lixia Guan, Bo Pan, Junyi Gao, Yifei Geng, Miao-Miao Niu, Zhiqin Li, Jindong Li

**Affiliations:** ^1^ Department of Pharmacy, The Hospital Affiliated to Medical School of Yangzhou University (Taizhou People’s Hospital), Taizhou, China; ^2^ Department of Pharmaceutical Analysis, China Pharmaceutical University, Nanjing, China; ^3^ Taizhou School of Clinical Medicine, The Affiliated Taizhou People’s Hospital of Nanjing Medical University, Taizhou, China

**Keywords:** monopolar spindle 1 (MPS1), histone deacetylase 8 (HDAC8), hepatocellular carcinoma (HCC), dual-targeted inhibitors, structure-based virtual screening

## Abstract

**Background:**

Overexpression of monopolar spindle 1 (MPS1) and histone deacetylase 8 (HDAC8) is associated with the proliferation of liver cancer cells, so simultaneous inhibition of both MPS1 and HDAC8 could offer a promising therapeutic approach for the treatment of liver cancer. Dual-targeted MPS1/HDAC8 inhibitors have not been reported.

**Methods:**

A combined approach of pharmacophore modeling and molecular docking was used to identify potent dual-target inhibitors of MPS1 and HDAC8. Enzyme inhibition assays were performed to evaluate the optimal compound with the strongest inhibitory activity against MPS1 and HDAC8. The selectivity of MPH-5 for MPS1 and HDAC8 was assessed on a panel of 68 kinases and other histone deacetylases. Subsequently, molecular dynamics (MD) simulation verified the binding stability of the optimal compound to MPS1 and HDAC8. Ultimately, *in vitro* cellular assays and *in vivo* antitumor assays evaluated the antitumor efficacy of the most promising compound for the treatment of hepatocellular carcinoma.

**Results:**

Six dual-target compounds (MPHs 1–6) of both MPS1 and HDAC8 were identified from the database using a combined virtual screening protocol. Notably, MPH-5 showed nanomolar inhibitory effect on both MPS1 (IC_50_ = 4.52 ± 0.21 nM) and HDAC8 (IC_50_ = 6.07 ± 0.37 nM). MD simulation indicated that MPH-5 stably binds to both MPS1 and HDAC8. Importantly, cellular assays revealed that MPH-5 exhibited significant antiproliferative activity against human liver cancer cells, especially HepG2 cells. Moreover, MPH-5 exhibited low toxicity and high efficacy against tumor cells, and it overcomes drug resistance to some extent. In addition, MPH-5 may exert its antitumor effects by downregulating MPS1-driven phosphorylation of histone H3 and upregulating HDAC8-mediated K62 acetylation of PKM2. Furthermore, MPH-5 showed potent inhibition of HepG2 xenograft tumor growth in mice with no apparent toxicity and presented favorable pharmacokinetics.

**Conclusion:**

The study suggests that MPH-5 is a potent, selective, high-efficacy, and low-toxicity antitumor candidate for the treatment of hepatocellular carcinoma.

## 1 Introduction

Hepatocellular carcinoma (HCC) is the most common type of primary liver cancer and one of the major malignancies with high cancer mortality rates worldwide (Addissouky et al., 2024). Despite significant advances in the treatment of HCC, the majority of patients with advanced HCC continue to experience drug resistance and disease progression (Yang et al., 2024). Most patients are not suitable for surgical resection or liver directed therapeutic due to advanced symptom presentation (Chan et al., 2023). In addition, systemic therapies are mostly ineffective in achieving long-term survival (Reig et al., 2022). Furthermore, the pace of new drug development is very slow. Between the years 2007 and 2016, only one drug (sorafenib), was approved for treating advanced HCC (Grieb et al., 2019). Although sorafenib extends the overall survival (OS) of patients with advanced hepatocellular carcinoma (HCC) to 11 months, its efficacy is confined to selected individuals and is associated with serious side effects, as well as being very expensive (Kant et al., 2021; Jiang et al., 2023). Importantly, the emergence of drug resistance has become a major obstacle to the clinical management of patients with HCC (Jiang et al., 2023). Therefore, there is an urgent need to find new drugs for the treatment of HCC in order to control disease progression.

**FIGURE 1:**
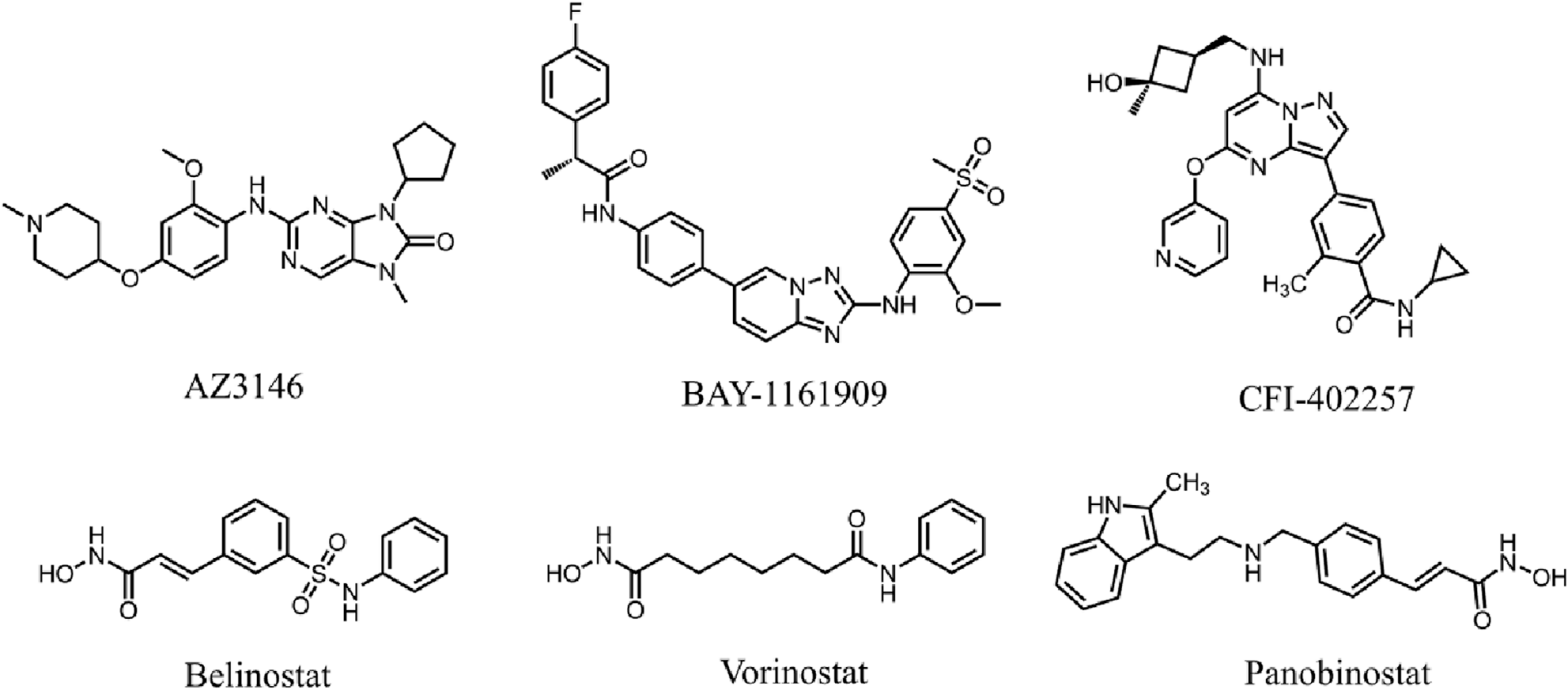
Reported MPS1 and HDAC8 inhibitors.

Monopolar spindle 1 (MPS1), was originally discovered in budding yeast cells as a dual-specificity protein kinase that phosphorylates tyrosine and threonine (Lauzé et al., 1995). As an upstream component of spindle assembly checkpoint (SAC), MPS1 is essential for the initiation and inhibition of SAC signaling (Gui et al., 2020). SAC is a signalling cascade that functions to detect chromosome misorientation and segregation errors (Xie et al., 2017). Consequently, MPS1 is essential for correct chromosome alignment, orientation, and segregation during mitosis (Wengner et al., 2016; Maciejowski et al., 2017). It has been shown that MPS1 was over-expressed during mitosis in cancer cell lines (Carter et al., 2006). MPS1 promotes the proliferation and migration of HCC cells, and MPS1 knockdown inhibits cell growth and colony formation in HCC (Carter et al., 2006; Liu et al., 2015). In addition, *in vivo* silencing of MPS1 limited the intrahepatic spread of HCC tumors in the liver (Miao et al., 2016). Based on these findings, MPS1 is expected to be a novel target for the treatment of HCC. Several MPS1 inhibitors have been developed and have undergone preclinical assessments in recent times, such as AZ3146, BAY-1161909, and CFI-402257 (Figure 1) (Bavetsias and Linardopoulos, 2015; Hewitt et al., 2010; Zeng et al., 2023). Despite the progress made with MPS1 inhibitors, certain drawbacks in terms of toxicity and drug resistance cannot be ignored (Zeng et al., 2023). Studies have shown that AZ3146 induces drug-specific MPS1 point mutations, which can lead to resistance (Gurden et al., 2015).

Histone deacetylases (HDAC) are a crucial class of epigenetic enzymes that catalyze the deacetylation of histones and non-histone proteins (West and Johnstone, 2014). HDAC inhibitors (HDACis) regulate the cell cycle and induce apoptosis by inhibiting abnormal HDAC activity (Pandolfi, 2001). The HDAC8 isoform, a class 1 HDAC, consists of 377 amino acids with a molecular weight of approximately 42 kDa (Rajaraman et al., 2023). Dysregulation of HDAC8 expression has been shown to be closely associated with HCC tumourigenesis (Tao et al., 2022). It has been revealed that the expression of HDAC8 is significantly upregulated in both HCC cell lines and tumor tissues (Wu et al., 2013). Inhibition of HDAC8 significantly suppresses the proliferative and migratory activities of HCC cells (Wang et al., 2017). To date, FDA has approved several HDAC inhibitors, including Belinostat, Vorinostat, and Panobinostat (Figure 1), which have shown significant therapeutic activity in hematological tumors but weak activity in solid tumors (Qiu et al., 2013; Srinivas, 2017). In clinical practice, these inhibitors have similar dose-limited toxicity, and only exhibit acceptable safety and tolerability in certain hematological diseases (Ho et al., 2020). Belinostat is associated with genotoxicity and dose-limiting toxicities such as fatigue, atrial fibrillation, diarrhoea, nausea and vomiting in the treatment of certain solid tumors (Lee et al., 2015; Cashen et al., 2012). Given the limited efficacy and low homologous selectivity, it is particularly important to develop more effective and less toxic HDAC8 inhibitors.

Aberrant expression of MPS1 affects the function of SAC to detect misdirected chromosomes, leading to abnormal cell proliferation and division (Liu et al., 2015). HDAC8 is capable of modifying chromatin structure by deacetylating histones, and thus plays a pivotal role in the regulation of gene expression, cell proliferation, migration, differentiation and metastasis (Peng et al., 2020). Considering that MPS1 and HDAC8 are closely associated with the formation of chromosomal structures and both are overexpressed in HCC tumors, dual inhibition of MPS1 and HDAC8 may provide a novel strategy for the treatment of HCC. Currently, the inherent challenges of toxicity and resistance associated with MPS1 and HDAC8 inhibitors are increasingly emerging as significant concerns. Studies have shown that combination therapy of MPS1 inhibitors with drugs such as paclitaxel has the potential to enhance tumor inhibition (Maia et al., 2018; Atrafi et al., 2021). Meanwhile, it has been shown that HDAC8 inhibitors can effectively inhibit FLT3-ITD^+^ AML cells in combination with FLT3 inhibitors (Long et al., 2020). Although MPS1 inhibitors and HDAC8 inhibitors have favorable therapeutic effects when combined with other drugs, the combination usually results in non-overlapping resistance mechanisms and different toxicities (de Lera and Ganesan, 2016). Dual-target inhibitors can exert the same favorable effects as drug combination therapies while avoiding their adverse risks to some extent (Bayat Mokhtari et al., 2017; Fu et al., 2017). Therefore, we aim to develop low-toxicity and efficient dual-target MPS1/HDAC8 inhibitors as antitumor agents. To the best of our knowledge, MPS1/HDAC8 dual targeted inhibitors have been rarely reported so far.

Structure-based virtual screening is a computational method employed in early-stage drug discovery to identify novel inhibitors from a chemical compound library targeting specific proteins (Li and Shah, 2017). Molecular docking predicts the optimal conformation of small molecule ligands within the active sites of target proteins and quantifies the energetics of intermolecular interactions (Meng et al., 2011). Pharmacophore model screening identifies compounds with characteristics that confer activity against the target (Li et al., 2022). The integrated application of molecular docking and pharmacophore modeling may effectively identify lead compound. In prior studies, we successfully identified potent dual-targeting inhibitors through structure-based virtual screening (Zhou et al., 2022; Zheng et al., 2021). In this study, we developed a novel dual-targeted MPS1/HDAC8 inhibitor (MPH-5) using a combined virtual screening protocol. The binding stability of MPH-5 at the active sites of MPS1 and HDAC8 was confirmed by MD simulation. Additionally, MPH-5 showed excellent antitumor efficacy *in vitro* and *in vivo*. Therefore, dual-targeting MPS1/HDAC8 inhibitors may offer a novel therapeutic strategy for HCC treatment.

## 2 Materials and methods

### 2.1 Cell culture and materials

All cells were obtained from Cell Bank of the Chinese Academic of Sciences (Shanghai, China). All the cell lines were incubated at 37°C in a humidified atmosphere of 5% CO_2_ and 95% air. Hit compounds were purchased from WuXi AppTec (Shanghai, China). MPS1 and HDAC8 proteins were obtained from Abcam (Cambridge, MA, United States).

### 2.2 Pharmacophore construction

The crystal structures of MPS1 (PDB ID: 4C4E) and HDAC8 (PDB ID: 1T64) proteins were obtained from the Protein Data Bank (PDB), and then were imported into the Molecular Operating Environment (MOE), respectively. First, both crystal structures were optimized using the QuickPrep tool of MOE, including energy minimization, removal of unbound water, calculation of partial charges, and addition of polar hydrogen. Then, structure-activity relationships were analyzed based on the above structure of the MPS1 protein by the Ligand Interaction tool in MOE. Based on structure-activity relationship analysis, the pharmacophore features of MPS1 were constructed, including hydrogen bond acceptors, hydrogen bond donors, and hydrophobic centroid.

### 2.3 Virtual screening

A database of 35,000 compounds was constructed through the application of combinatorial chemistry, then converted into 3D structures using MOE’s energy minimization program. Pharmacophore screening was performed in this database based on the MPS1 pharmacophore models constructed above. Subsequently, the screened compounds were subjected to further screening for docking based on the structures of MPS1 and HDAC8. The Dock tool in MOE was used to dock each compound into the active site of MPS1 and HDAC8. Docking was performed using the Triangle Matcher method and the London dG scoring algorithm. A low molecular docking energy score correlates with high binding affinity.

### 2.4 *In vitro* MPS1 inhibitory assay

The methodology is as previously described (Naud et al., 2013). In brief, the experiment was conducted using 384-well black low-volume plates, which were loaded with MPS1 (concentration range of 3–12.5 nM), 5 μM fluorescence-labelled peptide (sequence: 5FAM-DHTGFLTEYVATR-CONH2), 10 μM ATP, 1% (v/v) DMSO or the test compound (concentrations in 1% (v/v) DMSO ranged from 0.25 to 100 μM), and assay buffer. Conducted at room temperature for 60 min, the reaction was ceased upon the incorporation of 0.1 M HEPES-buffered saline, complete with 20 mM EDTA and 0.05% (v/v) Brij-35. The plate was read on a Caliper EZ reader II (PerkinElmer Life Sciences, Waltham, MA, United States). IC_50_ values were determined after testing the compounds in the concentration range of 0.25 nM to 100 μM.

### 2.5 *In vitro* HDAC8 inhibitory assay

The methodology is consistent with that previously described (Senger et al., 2016). Inhibition of HDAC8 was determined in 96-well plates. The 22.5 μL of HDAC8 enzyme in incubation buffer (50 mM KH_2_PO_4_, 15 mM Tris, pH 7.5, 3 mM MgSO_4_·7H_2_O, 10 mM MgSO_4_) was mixed with 2.5 μL of inhibitor in DMSO and 5 μL of Z-L-Lys(ε-trifluoroacetyl)-AMC (150 μM), and incubated for 90 min at 37°C. Then 30 µL of stop solution (33 µM trichostatin A (TSA) and 6 mg/mL trypsin in trypsin buffer) was added, and the plate was incubated again at 37°C for 30 min. The assessment of fluorescence intensity was executed at 390 nm and 460 nm via a microplate reader.

### 2.6 *In vitro* selectivity assay

The inhibitory effects of compound MPH-5 on a panel of 68 kinases and other histone deacetylases were evaluated to assess the selectivity profile of MPH-5. This experiment was conducted by ICE Bioscience Inc. (Beijing, China).

### 2.7 MD simulation

The structures of MPS1 (PDB ID: 4C4E) and HDAC8 (PDB ID: 1T64) were obtained from the PDB. MD simulation was performed using GROMACS (version 2021.5) to analyze binding stability. First, The MPH-5 topology file was obtained under the GAFF force field from the Acpype Server (www.bio2byte.be). MPS1 and HDAC8 proteins were topologised under the AMBER99SB-ILDN force field, respectively. Then the ligand and protein files were combined to form a complex system. The system was encapsulated in a cubic simulation volume of 1.0 nm, solvated using the SPC/E water model and neutralised with Na^+^ and Cl^−^. Next, the optimization of the system’s energy state was conducted through a 5,000-step steepest descent algorithmic procedure, ensuring a robust minimization outcome. The V-rescale thermostat was used to perform NVT simulation to keep the temperature at 300 K. The simulation was performed under NPT conditions using the Parinello-Rahman barostat to maintain an equilibrium pressure of 1 bar throughout the simulation period. Finally, 50 ns MD simulations were performed for the MPH-5-MPS1 and MPH-5-HDAC8 systems. These data were processed using GraphPad Prism 6.0 software.

### 2.8 *In vitro* cytotoxicity assay

The cells (5 × 10^4^ cells/well) were incubated overnight in 96-well plates. A series of solutions containing varying concentrations of inhibitors were added and incubated at 37°C for a period of 72 h. Subsequently, the culture medium was removed, followed by a subsequent incubation of the cells with a 5 mg/mL MTT stock solution for 4 h. After centrifugation, the precipitate was solubilized in DMSO and gently shaken for 15 min. Finally, the absorbance measurement at 570 nm was completed using a microplate reader. Data analysis was performed using GraphPad Prism 6.0 software.

### 2.9 Analysis of gene expression

Total RNA was extracted after the indicated treatments using the RNeasy Mini Kit (cat. no. 74106; QIAGEN, Hilden, Germany) following the manufacturer’s protocol. The concentration of RNA was measured on a plate reader, and then the extracted RNA was converted to cDNA using a High-Capacity RNA-to-cDNA kit from Applied Biosystems (Thermo Fisher Scientific, Inc.). Quantitative real-time polymerase chain reaction was performed in triplicate in a 96-well optical PCR plate using a QuantStudio Real-Time PCR System. Gene expression levels were evaluated using the comparative CT method. Graphical representations of the data were generated by GraphPad Prism 8.

### 2.10 *In vivo* antitumor assay

Male BALB/c mice (6 weeks old) were purchased from Changzhou Cavens Experimental Animal Limited Company (Changzhou, China). The xenograft model was established by subcutaneous injection of a suspension of HepG2 human liver cancer cells in PBS (200 μL, 1 × 10^7^ cells) into the mice. The mice were randomized into four groups and received intraperitoneal injections of vehicle or MPH-5 at concentrations of 1, 5 and 10 mg/kg. Tumor volume was measured at 3-day intervals and calculated as follows: (c × c × d)/2 (c, the smallest diameter; d, the largest diameter). The experiments involving animals were approved by the Ethics Committee of China Pharmaceutical University.

### 2.11 *In vivo* pharmacokinetic studies

MPH-5 was administered intraperitoneally to BALB/c mice at a dose of 5 mg/kg. The blood samples (0.25 mL) were collected at 0.25, 0.5, 1, 2, 4, 8, 16 and 24 h after dosing and centrifuged to obtain the plasma fraction. Plasma samples (50 μL) were transferred into a 96-well plate, followed by the addition of an internal standard solution of methanol/acetonitrile (1:1, v/v) (200 μL). After vortexing for 5 min, the mixture was centrifuged (12,000 rpm for 5 min) to obtain the supernatant (60 μL). A liquid chromatography-tandem mass spectrometry (LC-MS/MS) system was used for the pharmacokinetic study. Data were processed using Phoenix software.

## 3 Results and discussion

### 3.1 Establishment of the MPS1 pharmacophore model

**FIGURE 2:**
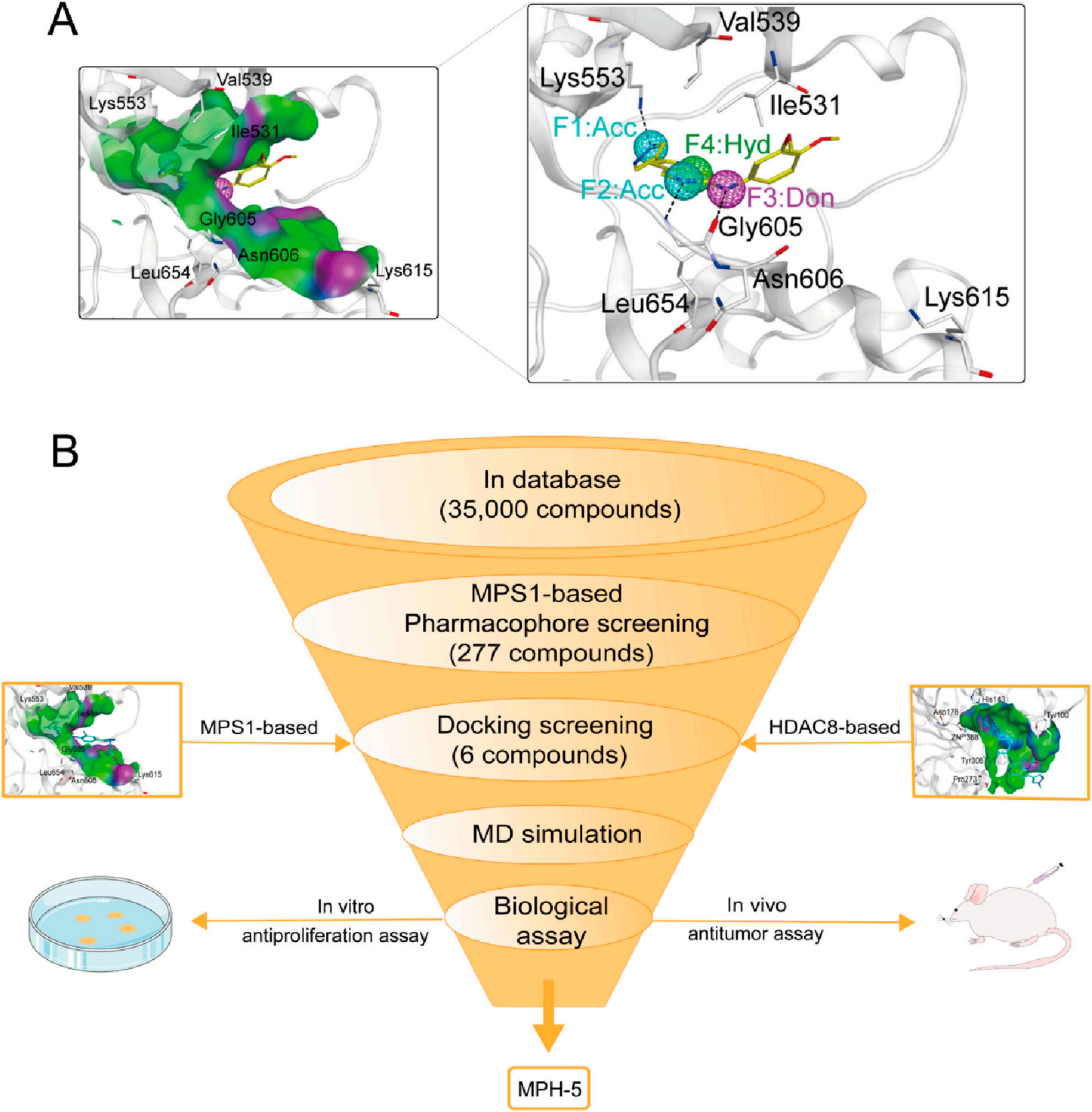
**(A)** The pharmacophore models based on the MPS1 structure. **(B)** The multi-step virtual screening workflow.

Pharmacophore modeling can generate molecular recognition features at the 3D level to ensure optimal ligand-receptor binding modes (Yang, 2010). We obtained the crystal structure of MPS1 (PDB ID: 4C4E) in complexed with the original ligand by PDB database, and generated pharmacophore models of MPS1 using Pharmacophore Query Editor of the MOE. As shown in Figure 2A, the two Acc features corresponded to hydrogen bonds formed with Lys553, and Gly605. The Don feature corresponded to a hydrogen bond formed with the oxygen atom of Gly605 residue. The Hyd feature corresponded to hydrophobic interactions formed with the Val539, Ile531 and Leu654 residue. Thus, the final pharmacophore models contained four features, including two hydrogen bond acceptor features (F1 and F2: Acc, cyan color), a hydrogen bond donor feature (F3: Don, purple color) and a hydrophobic centroid feature (F4: Hyd, green color).

### 3.2 Virtual screening

**FIGURE 3:**
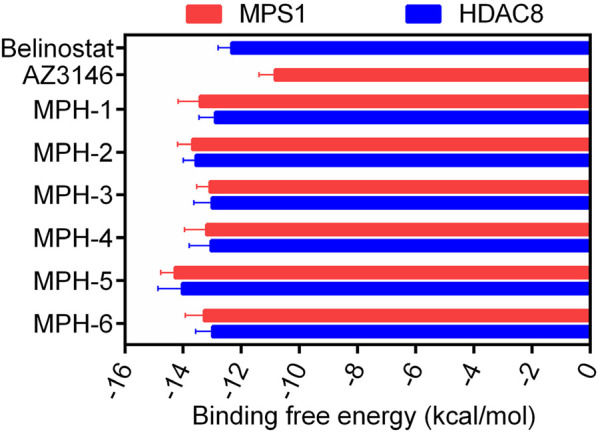
The binding free energy (kcal/mol) of six hits (MPHs 1–6).

**FIGURE 4:**
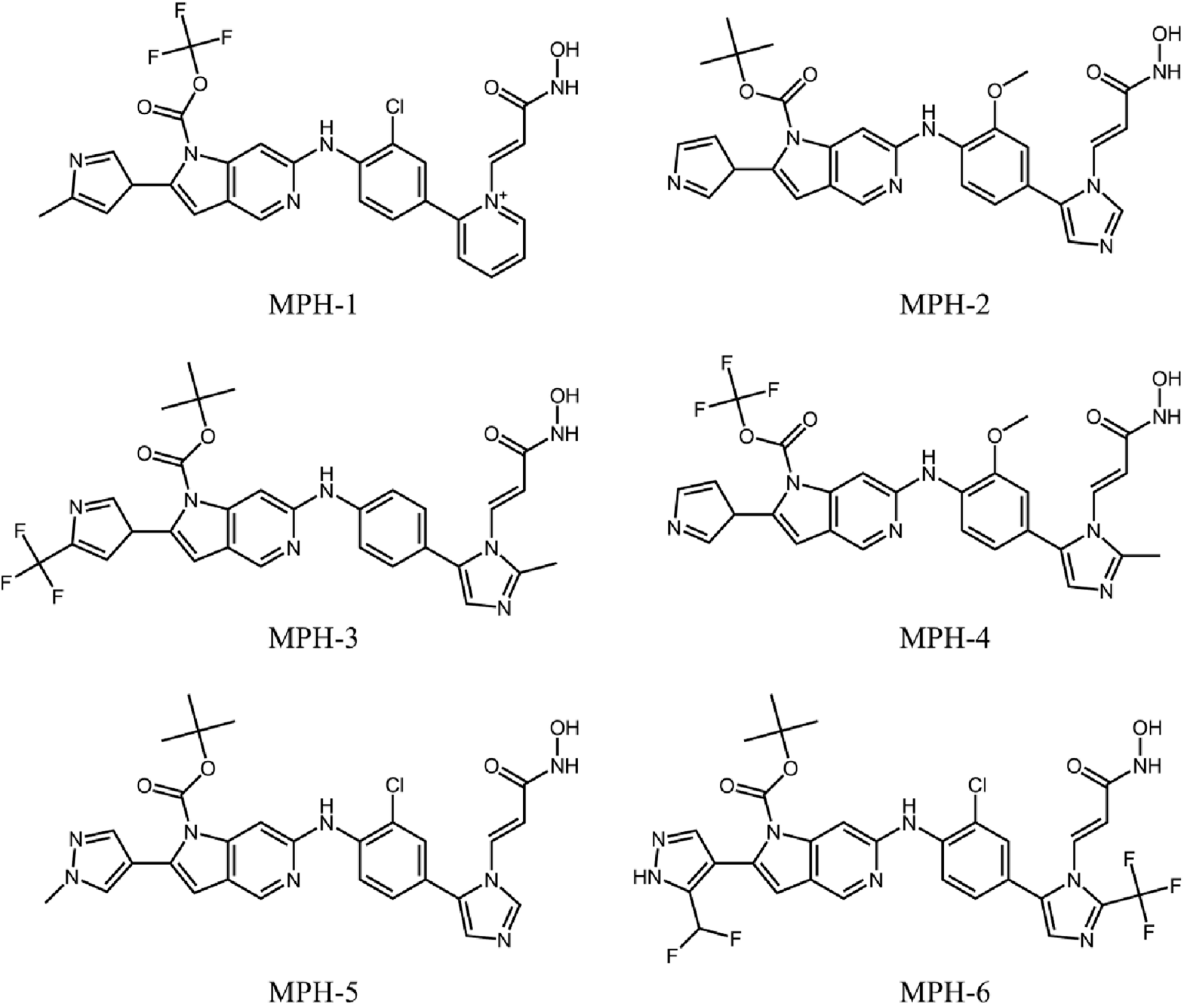
The chemical structures of six hits (MPHs 1–6).

The flowchart of the virtual screening is depicted in Figure 2B. High-resolution structures of MPS1 (PDB ID: 4C4E) and HDAC8 (PDB ID: 1T64) were obtained from the PDB database. Constructed an in-house database containing 35,000 compounds and converted the structures into three-dimensional structures. Based on the pharmacophore features created above, the compounds matching the pharmacophore features were retrieved from the in-house database. A smaller root-mean-square deviation (RMSD) value indicates a stronger interaction force and higher affinity between the ligands and the receptors. Through MPS1-based pharmacophore screening, we screened 277 compounds matching the pharmacophore features. Then the 277 compounds were subjected to MPS1-based molecular docking screen. The docking score was used to assess the binding affinity of a compound to MPS1; the lower the score, the stronger the affinity. The cutoff value was established using a docking score of −10.84 kcal/mol for the control compound AZ3146. We selected 93 compounds with docking score below −10.84 kcal/mol; and then, the 93 compounds were further docked to the active site of HDAC8. The docking score of the control compound, belinostat, was recorded as −12.33 kcal/mol, with 39 compounds having docking scores below this threshold. Finally, the six compounds (MPHs 1–6) with the lowest ranked docking scores were obtained for further evaluation of enzyme inhibitory activity. Docking scores and structures of MPHs 1–6 are shown in Figures 3, 4, respectively.

### 3.3 Interaction analysis

**FIGURE 5:**
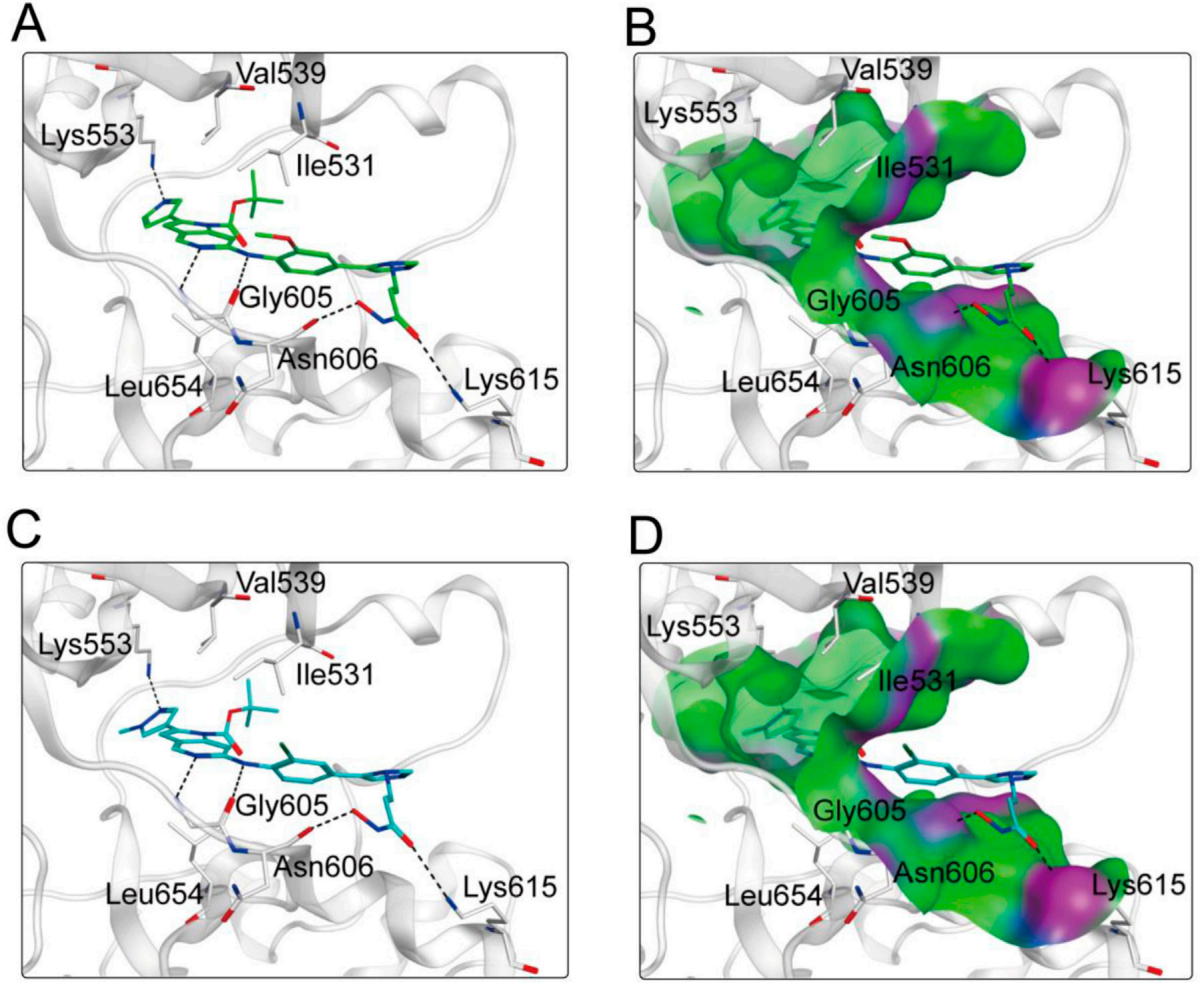
**(A, B)** The binding mode of MPH-2 (green sticks) in the active site of MPS1. **(C, D)** The binding mode of MPH-5 (cyan sticks) in the active site of MPS1. Residues in the active site are represented by gray sticks. The hydrogen bonds are shown as black dashed lines.

**FIGURE 6:**
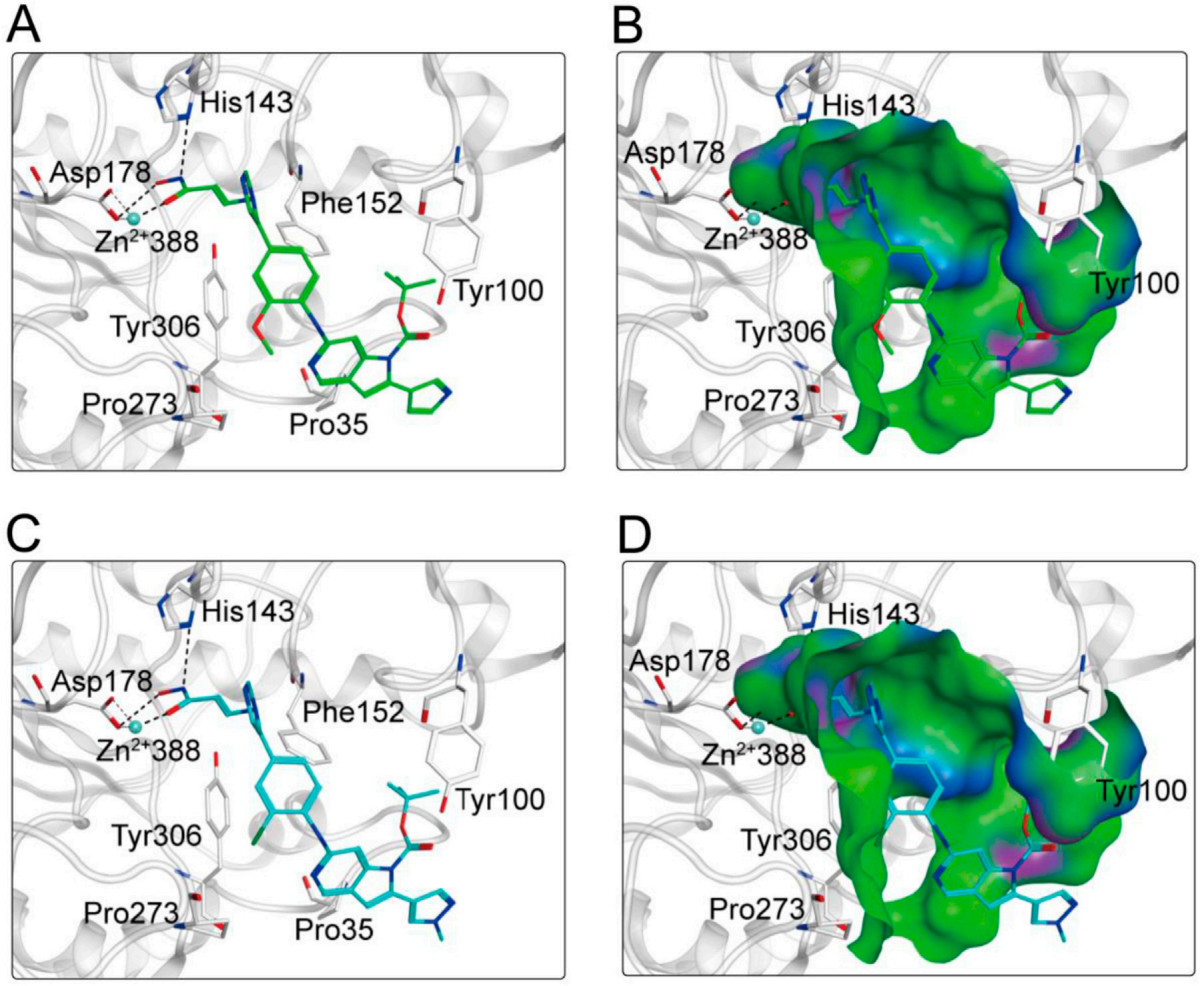
**(A, B)** The binding mode of MPH-2 (green sticks) in the active site of HDAC8. **(C, D)** The binding mode of MPH-5 (cyan sticks) in the active site of HDAC8. Residues in the active site are represented by gray sticks. The hydrogen bonds are shown as black dashed lines.

Based on the above docking results, it was found that MPH-2 and MPH-5 had the lowest docking scores. Therefore, we further investigated the binding modes of MPH-2 and MPH-5 with MPS1 and HDAC8, respectively. The binding mode and binding surface map of MPH-2 (green sticks) and MPH-5 (cyan sticks) docked to MPS1 protein are shown in Figure 5. MPH-2 and MPH-5 formed hydrogen bonding with amino acids Lys553 and Gly605, matching the Acc feature (F1 and F2) and Don feature (F3). In addition, MPH-2 and MPH-5 created hydrogen bonds with Asn606, Lys615, which anchored the direction of the binding process. In addition, hydrophobic interactions between MPH-2 and MPH-5 with the hydrophobic amino acids Val539, Ile531 and Leu654 stabilize them in the hydrophobic pocket of MPS1. As seen in the binding surface map, MPH-2 and MPH-5 nicely occupied the pocket of MPS1. Figure 6 shows the binding mode and binding surface map of MPH-2 and MPH-5 docked to HDAC8 protein. The hydroxamic acid groups of MPH-2 and MPH-5 extended into the interior of the HDAC8 pocket to form ionic bonds with Zn ions and hydrogen bonds with His143 and Asp178, contributing to the stabilization of MPH-2 and MPH-5 at the HDAC8 binding site. In addition, MPH-2 and MPH-5 formed hydrophobic interactions with the hydrophobic amino acids Phe152, Pro53, and Pro273. Thus, the results of the docking modes described above suggest that MPH-2 and MPH-5 bind stably at the active sites of both MPS1 and HDAC8.

### 3.4 Inhibitory effects on MPS1 and HDAC8

Subsequently, we conducted enzyme activity inhibition experiments to test the inhibitory effects of MPHs 1–6 on MPS1 and HDAC8. As shown in Table 1, MPHs 1–6 showed nanomolar inhibitory activities against both MPS1 and HDAC8. MPS1 inhibitor AZ3146 and HDAC8 inhibitor belinostat were used as positive controls. The IC_50_ values of all the six compounds were lower than that of the positive control, respectively. AZ3146 showed an IC_50_ value of 34.35 ± 2.87 nM, whereas there was no inhibitory effect on HDAC8. Belinostat exhibited inhibitory activity against HDAC8 (IC_50_ = 22.17 ± 1.43 nM) and no inhibition on MPS1. Among them, MPH-5 had the strongest inhibitory effect. Its IC_50_ values for MPS1 (IC_50_ = 4.52 ± 0.21 nM) and HDAC8 (IC_50_ = 6.07 ± 0.37 nM) were about 8-fold higher than that of AZ3146 and about 4-fold higher than that of belinostat, respectively. As MPH-5 showed the most potent inhibitory effects on MPS1 and HDAC8, a series of selectivity assays were performed on a panel of 68 kinases and other histone deacetylases to further explore the selectivity effects of MPH-5. The results showed that MPH-5 did not significantly inhibit these kinases and other histone deacetylases with IC_50_ > 10 μM (Table 2). These data suggest that MPH-5 specifically binds to MPS1 and HDAC8 to exert its inhibitory activity. In addition, MPH-5 displayed the lowest docking scores. Thus, these results of inhibitory activity are also consistent with the results of molecular docking studies.

**TABLE 1:** The inhibitory effects of MPHs 1–6 on MPS1 and HDAC8.

Compounds	MPS1 (IC_50_, nM)	HDAC8 (IC_50_, nM)
MPH-1	9.38 ± 0.41	17.52 ± 2.32
MPH-2	7.60 ± 0.35	8.15 ± 0.58
MPH-3	13.25 ± 0.82	15.2 ± 1.94
MPH-4	11.74 ± 0.95	13.5 ± 0.61
MPH-5	4.52 ± 0.21	6.07 ± 0.37
MPH-6	10.84 ± 0.69	16.62 ± 2.06
AZ3146	34.35 ± 2.87	No inhibition
Belinostat	No inhibition	22.17 ± 1.43

**TABLE 2:** Selectivity testing of MPH-5 on a panel of 68 kinases and other histone deacetylases.

Target	IC_50_ (μM)	Target	IC_50_ (μM)	Target	IC_50_ (μM)
ABL1	>10	FES	>10	LTK	>10
ABL2	>10	FGFR1	>10	LYN	>10
AXL	>10	FGFR2	>10	MERTK	>10
BLK	>10	FGFR3	>10	MET	>10
BMX	>10	FGFR4	>10	MST1R	>10
BTK	>10	FGR	>10	MUSK	>10
CSF1R	>10	FRK	>10	NTRK1	>10
CDK2	>10	FYN	>10	NTRK2	>10
DDR1	>10	PIM1	>10	NTRK3	>10
DDR2	>10	RAF1	>10	PDGFRA	>10
ALK	>10	ROS1	>10	PDGFRB	>10
EPHA1	>10	ZAK	>10	PTK2	>10
EPHA2	>10	TYRO3	>10	PTK2B	>10
EPHA3	>10	YES1	>10	PTK6	>10
EPHA4	>10	ZAP70	>10	RET	>10
EPHA5	>10	HCK	>10	ROS1	>10
EPHA6	>10	IGF1R	>10	SRC	>10
EPHA7	>10	INSR	>10	SYK	>10
EPHA8	>10	INSRR	>10	HDAC1	>10
EPHB1	>10	ITK	>10	HDAC2	>10
EPHB2	>10	JAK1	>10	HDAC3	>10
EPHB3	>10	JAK2	>10	HDAC4	>10
EPHB4	>10	JAK3	>10	HDAC5	>10
ERBB2	>10	KDR	>10	HDAC6	>10
ERBB4	>10	KIT	>10	HDAC7	>10

### 3.5 MD simulation

**FIGURE 7:**
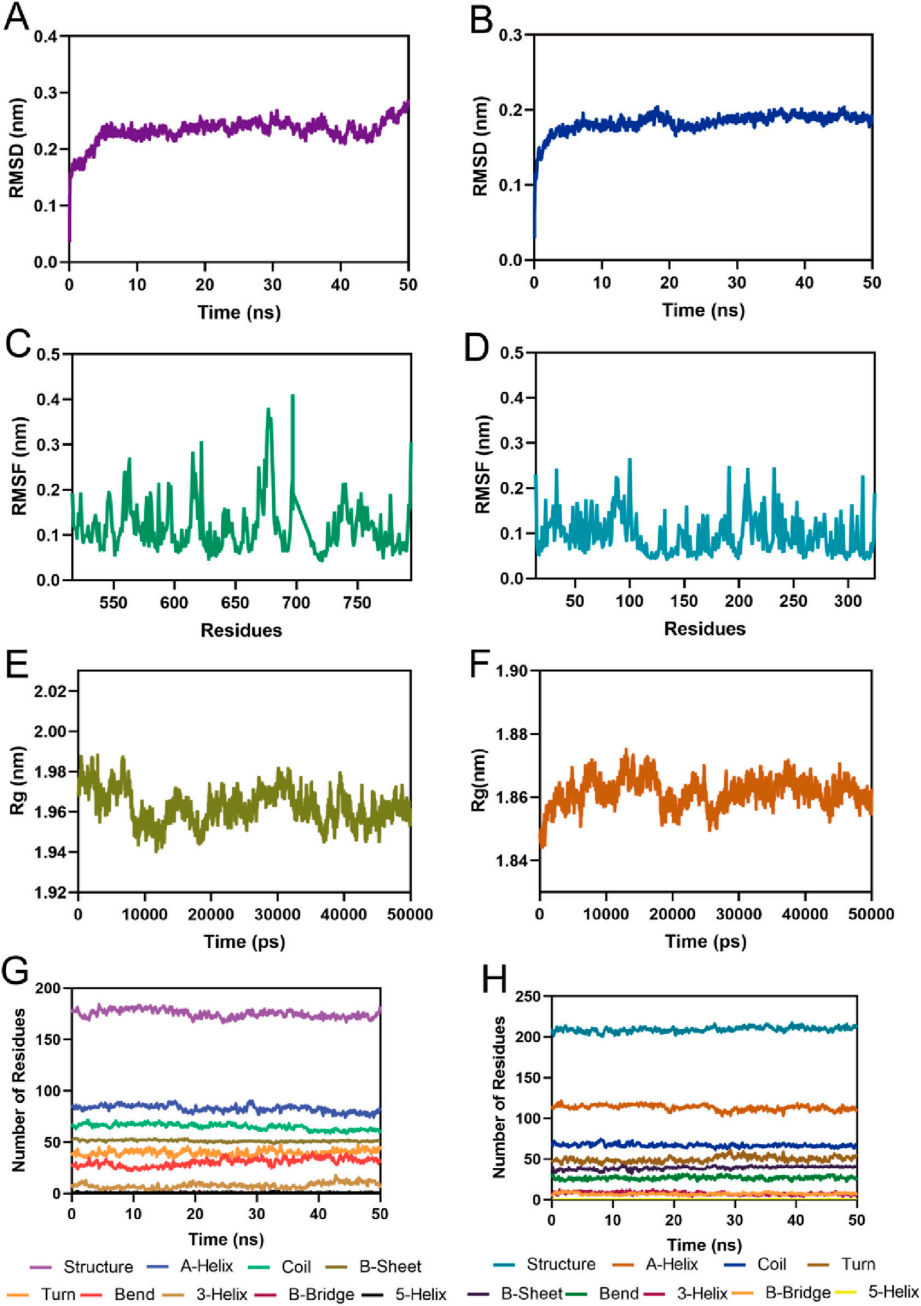
MD simulation of MPH-5 in complex with MPS1 and HDAC8. **(A, B)** The backbone RMSD for MPH-5 complexed with MPS1 and HDAC8, respectively. **(C, D)** The RMSF of the Cα atoms of MPS1 and HDAC8, respectively. **(E, F)** Radius of Gyration of MPS1 and HDAC8, respectively. **(G, H)** The secondary structures analysis of MPS1 and HDAC8, respectively.

To assess the binding stability of MPH-5 to MPS1 and HDAC8, the 50 ns MD simulation was performed using GROMACS. Fluctuations in root mean square deviation (RMSD) can indicate the stability of the complex during simulation. As shown in Figure 7A, the RMSD of the MPH-5-MPS1 complex stabilized after 5 ns and fluctuated slowly around 0.23 nm; in Figure 7B, the RMSD of the MPH-5-HDAC8 complex remained stable at around 0.18 nm after a slight increase. These data suggest that MPH-5 binds stably to MPS1 and HDAC8. In addition, the root mean square fluctuation (RMSF) reflects the flexibility of the amino acid movement during the simulation. In Figure 7C, the RMSF values of key residues Ile531, Val539, Lys553, Gly605, Asn606, Lys615, and Leu654 in the MPS1 active binding site were all less than 0.29 nm; In Figure 7D, the RMSF of key residues Pro53, His143, Phe152, Asp178, Pro273 in the active site of HDAC8 were shown to be below 0.16 nm, reflecting the stable binding of MPH-5 to MPS1 and HDAC8. In addition, Figures 7E, F) shows the radius of gyration (Rg) of MPS1 and HDAC8. The Rg values of MPS1 and HDAC8 were stable around 1.96 and 1.86 nM respectively, indicating that the proteins were structurally compact throughout the simulation. In Figures 7G, H, there were no obvious fluctuations in the secondary structure of the MPS1 and HDAC8 proteins, suggesting that the proteins remain structurally stable after binding to MPH-5. In conclusion, MPH-5 binds stably to the active sites of MPS1 and HDAC8, and remains structurally stable throughout the entire simulation process. Next, based on the combined stability of MPH-5 with MPS1 and HDAC8, *in vitro* and *in vivo* inhibition assays were performed to evaluate the inhibitory effect.

### 3.6 Cell growth inhibitory activity

Next, MTT assays were conducted to assess the antiproliferative activity of the six compounds (MPHs 1–6) on HepG2 liver cancer cells. As shown in Table 3, all MPHs 1–6 showed varying degrees of antiproliferative activity, among which MPH-5 was the most effective compound in inhibiting the growth of HepG2 cells (IC_50_ = 0.19 μM). Subsequently, we further investigated the cellular antiproliferative activity of MPH-5 on a series of human liver cancer cells. The results are shown in Table 4. MPH-5 exhibited significant antiproliferative activity in a series of drug-sensitive liver cancer cells (IC_50_ = 0.19 μM for HepG2, IC_50_ = 0.86 μM for BEL7402, and IC_50_ = 0.32 μM for Huh-1, and IC_50_ = 0.64 μM for Li-7). Specifically, MPH-5 showed the strongest cytotoxicity to HepG2 cell lines. Meanwhile, we assessed the efficacy of MPH-5 against drug-resistant liver cancer cells (R-HepG2). The data showed that MPH-5 had an inhibitory effect on R-HepG2 cells (IC_50_ = 0.27 μM), which was slightly lower than that of drug-sensitive HepG2 cells, and higher than that of other drug-sensitive liver cancer cells (Table 4). This indicated that MPH-5 had overcome drug resistance to some extent. To assess the safety of MPH-5, we performed cytotoxicity experiments on a series of normal cells (L02, HEK293, MCF10A, BEAS-2B). As shown in Table 5, MPH-5 exhibited no significant inhibitory effect on normal cells (IC_50_ > 10 μM), indicating that MPH-5 has low toxicity. The data demonstrate that MPH-5 has low toxicity and high efficacy in the treatment of cancer.

**TABLE 3:** The cytotoxicity of MPHs 1–6 on HepG2 liver cancer cells.

Name	IC_50_ (μM)^a^					
	MPH-1	MPH-2	MPH-3	MPH-4	MPH-5	MPH-6
HepG2	0.47	0.32	0.55	0.41	0.19	0.53

^a^ IC_50_ (μM) is the concentration of compound needed to reduce cell growth by 50% following 72 h cell treatment with MPHs 1–6.

**TABLE 4:** The cytotoxicity of MPH-5 on a series of drug-sensitive liver cancer cells and drug-resistant liver cancer cells (R-HepG2).

Name	IC_50_ (μM)^a^				
	HepG2	BEL7402	Huh-1	Li-7	R-HepG2
MPH-5	0.19	0.86	0.32	0.64	0.27

^a^ IC_50_ (μM) is the concentration of compound needed to reduce cell growth by 50% following 72 h cell treatment with MPH-5.

**TABLE 5:** The cytotoxicity of MPH-5 on a series of normal cells.

Name	IC_50_ (μM)^a^			
	L02	HEK293	MCF10A	BEAS-2B
MPH-5	>10	>10	>10	>10

^a^ IC_50_ (μM) is the concentration of compound needed to reduce cell growth by 50% following 72 h cell treatment with MPH-5.

### 3.7 Analysis of gene expression

**FIGURE 8:**
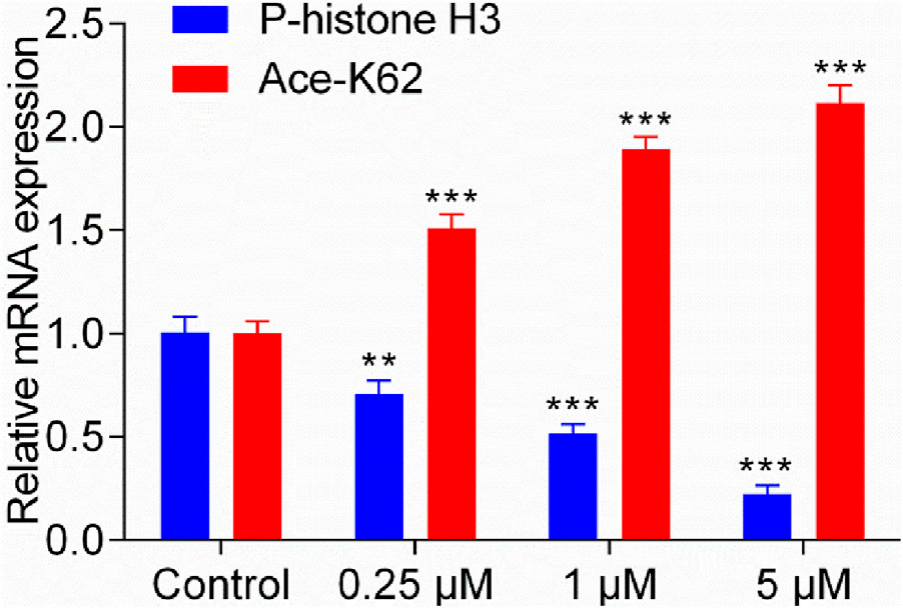
Effect of MPH-5 on gene expression levels in HepG2 cells. The gene expression of P-histone H3 and Ace-K62 was detected in HepG2 cells with the treatment of MPH-5 for 20 h (0, 0.25, 1, and 5 μM). **p < 0.01 vs. control. ***p < 0.001 vs. control. The results are represented as the mean ± SD, n = 3.

Previous studies indicate that MPS1 inhibitor exerts its antitumor effects by downregulating the levels of phosphorylated histone H3 (Naud et al., 2013), while HDAC8 inhibitor achieves its antitumor activity by upregulating lysine residue 62 acetylation (Ace-K62) levels of pyruvate kinase M2 (PKM2) (Zhang et al., 2020). The gene expression of P-histone H3 and Ace-K62 was detected in HepG2 cells with the treatment of MPH-5 for 20 h (at the concentration of 0, 0.25, 1, and 5 μM, respectively). As shown in Figure 8, compared to the control group, there was a dose-dependent regulation of phosphorylated histone H3 levels and K62 acetylation levels after 20 h. As the concentration of MPH-5 increases, the inhibitory effect on P-histone H3 and the promotional effect on Ace-K62 become more pronounced. This indicates that MPH-5 may exert its antitumor effect by downregulating MPS1-driven phosphorylation of histone H3 and upregulating HDAC8-mediated K62 acetylation of PKM2.

### 3.8 *In vivo* antitumor effect

**FIGURE 9:**
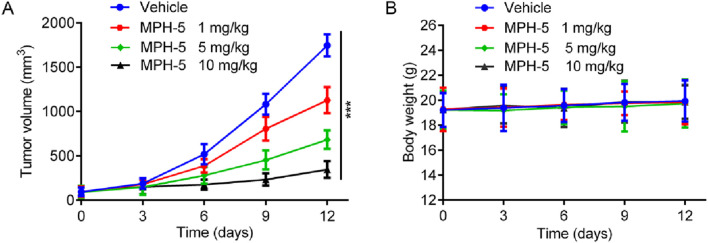
MPH-5 exhibited good antitumor activity to HepG2 cell-derived xenograft. **(A)** Changes in tumor volume. **(B)** Body weight of mice. Data are presented as the mean ± SD, n = 6. ***p < 0.001 means a significant difference versus the vehicle group.

Based on the excellent *in vitro* antiproliferative activity, we finally assessed the *in vivo* efficacy of MPH-5 in a HepG2 xenograft model. Mice were randomly assigned to four groups: vehicle control and MPH-5 at doses of 1, 5, and 10 mg/kg. As shown in Figure 9A, compared with the vehicle group, the MPH-5-treated group significantly and dose-dependently inhibited the tumor growth. In addition, MPH-5 did not induce any significant body weight change (Figure 9B), indicating that the MPH-5-treated groups did not cause severe systematic side effects. These results demonstrate that MPH-5 show significant antitumor activity in xenograft models, indicating its therapeutic potential.

### 3.9 *In vivo* pharmacokinetics

We further determined the pharmacokinetic profile for MPH-5 in BALB/c mice and the parameters are shown in Table 6. Following intraperitoneal (i.p.) injection of MPH-5 (5 mg/kg) into BALB/c mice, MPH-5 exhibited a prolonged elimination half-life (T_1/2_ = 3.46 h), high maximum plasma concentration (C_max_ = 2,921 nmol/L) and area under the drug-time curve (AUC = 13,765 h·nmol/L). Significantly, MPH-5 had achieved an encouraging level of bioavailability (F = 85%). Therefore, MPH-5 may have an ideal tumor-suppressing effect on the human body.

**TABLE 6:** Pharmacokinetic profile for MPH-5 in BALB/c mice.

Name	Route	Dose (mg/kg)	T_1/2_ (h)	C_max_ (nmol/L)	AUC (h·nmol/L)	*F* (%)
MPH-5	i.p.	5	3.46	2921	13,765	85

T_1/2_, elimination half-life; C_max_, maximum plasma concentration; AUC, area under the drug-time curve; *F*, bioavailability.

## 4 Conclusion

Currently, there is a concerning annual increase in the number of diagnosed liver cancer cases. Given the important role of MPS1 and HDAC8 in hepatocellular carcinoma, the development of effective MPS1/HDAC8 inhibitors is a novel approach to cancer treatment. In this study, we identified six novel dual-targeted MPS1/HDAC8 inhibitors (MPHs 1–6) from an in-house database of 35,000 compounds through structure-based virtual screening. Notably, MPH-5 demonstrated the most potent inhibitory activity against both MPS1 (IC_50_ = 4.52 ± 0.21 nM) and HDAC8 (IC_50_ = 6.07 ± 0.37 nM). Additionally, MD simulation supported the stable binding of MPH-5 to both MPS1 and HDAC8. Importantly, MPH-5 displayed significant antiproliferative activity against human liver cancer cells, featuring low toxicity and low drug resistance. In addition, MPH-5 may exert its antitumor effects by downregulating MPS1-driven phosphorylation of histone H3 and upregulating HDAC8-mediated K62 acetylation of PKM2. Moreover, *in vivo* experiments demonstrated that MPH-5 had an excellent inhibitory effect on HepG2 xenograft tumor. Notably, the concordance between biological assay outcomes and docking scores validated the structure-based virtual screening approach for predicting lead compound activity. In conclusion, MPH-5 emerges as a promising dual-targeted MPS1/HDAC8 inhibitor with potential for the treatment of hepatocellular carcinoma.

## Data Availability

The original contributions presented in the study are included in the article/supplementary material, further inquiries can be directed to the corresponding authors.
